# Antibodies against angiotensin II type 1 and endothelin-1 type A receptors are associated with microvascular obstruction after revascularized ST-elevation myocardial infarction

**DOI:** 10.1093/ehjopen/oeae099

**Published:** 2024-12-03

**Authors:** Giovanni Civieri, Laura Iop, Emanuele Cozzi, Sabino Iliceto, Francesco Tona

**Affiliations:** Cardiology Division, Department of Cardiac, Thoracic, Vascular Sciences and Public Health, University of Padua, Via N. Giustiniani 2, Padova 35128, Italy; Translational Biomedicine Group, Department of Cardiac, Thoracic, Vascular Sciences and Public Health, University of Padua, Via N. Giustiniani 2, Padova 35128, Italy; Transplant Immunology Unit, Department of Cardiac, Thoracic, Vascular Sciences and Public Health, University of Padua, Via N. Giustiniani 2, Padova 35128, Italy; Cardiology Division, Department of Cardiac, Thoracic, Vascular Sciences and Public Health, University of Padua, Via N. Giustiniani 2, Padova 35128, Italy; Cardiology Division, Department of Cardiac, Thoracic, Vascular Sciences and Public Health, University of Padua, Via N. Giustiniani 2, Padova 35128, Italy

ST-elevation myocardial infarction (STEMI) is a common cardiac emergency that causes considerable morbidity and mortality worldwide, characterized by complete occlusion of an epicardial coronary artery. Primary percutaneous coronary intervention (PPCI) allows the restoration of epicardial coronary blood flow. The resumption of epicardial coronary artery flow after STEMI, however, is not always a synonym for myocardial reperfusion. This phenomenon, known as no reflow, involves coronary microvascular obstruction (MVO), a condition of extreme microvascular dysfunction, as its pathophysiological substrate and is associated with poor outcomes.^1^

We have recently demonstrated that autoantibodies targeting a specific G-protein coupled receptor (GPCR), namely endothelin-1 type A receptor (ETAR), are associated with MVO after STEMI.^2^ We hypothesized that this association is driven by the pro-vasoconstrictive, pro-inflammatory, and pro-fibrotic properties of anti-ETAR autoantibodies (ETAR-AAs), which could contribute to MVO pathogenesis.^3^ However, we have also recently shown that another class of anti-GPCR autoantibodies, namely those targeting angiotensin II type 1 receptors (AT1R-AAs), is associated with left ventricular remodelling (LVR) and adverse prognosis after STEMI.^4^

The effects of AT1R-AAs on MVO have not been specifically investigated, nor have a possible cumulative effect of the two autoantibodies on MVO risk been investigated. To address these unanswered questions, we evaluated the differential association between autoantibody seropositivity and the prevalence of MVO in a cohort of timely revascularized STEMI patients who underwent cardiac magnetic resonance (CMR).

This prospective study was conducted at the Padua University Hospital between January 2022 and June 2023 and expands our previous findings:^2^ AT1R-AAs were measured on stored sera of previously included patients and six new patients were enrolled. Briefly, patients were eligible for study participation if they presented with STEMI and chest pain within 12 h of the first symptom onset. Cardiac magnetic resonance was performed with a 1.5-T scanner (Magnetom Avanto, Siemens Medical Solutions) using a comprehensive protocol as previously reported.^2^ Microvascular obstruction was defined as a hypoenhanced region within the infarcted myocardium.^5^ Serum levels of AT1R-AAs and ETAR-AAs were measured in all included patients. Blood samples were drawn within 12 h following PPCI. Autoantibodies targeting angiotensin II type 1 receptor and ETAR-AAs were determined by enzyme-linked immunosorbent technique (ELISA, CellTrend, Luckenwalde, Germany). Autoantibodies targeting angiotensin II type 1 receptor and ETAR-AAs seropositivity were defined according to the threshold concentration (>10 U/mL) provided by the manufacturer. Patients were then categorized into three mutually exclusive groups: double seronegative (seronegative for both AAs), single seropositive (seropositive for either AT1R-AAs or ETAR-AAs), or double seropositive (seropositive for both AAs).

Continuous data were presented as medians with interquartile ranges (IQRs) and compared using the Mann–Whitney *U* test or Kruskal–Wallis test, as appropriate. Categorical data were reported as frequencies with percentages and compared using the χ^2^ test or Fisher’s exact test, as appropriate. Predicted probabilities derived from univariate logistic regression analysis were also used to evaluate the correlation between continuous autoantibody levels and the probability of MVO. All tests were two-sided. Statistical significance was set at *P* < 0.05. Statistical analyses were performed using the statistical package R (version 4.3.1). The study protocol was approved by the local ethics committee (code number CESC 5478/AO/22) and all patients provided informed consent.

We recruited 56 patients, median age of 58.0 years (IQR: 53.2–68.7 years), 46 (82.1%) males. The baseline characteristics of the patients are shown in *Table 1*. Median time between STEMI and CMR was 21.0 days (8.7–28.0). Among the 56 patients, ETAR-AAs were seropositive in 22 (39.2%), while AT1R-AAs were seropositive in 16 (28.5%).

**Table 1:** Demographic and clinical characteristics of patients with autoantibodies double seronegativity, single, or double seropositivity

	All patients (*n* = 56)	Double seronegative (*n* = 31, 55.4%)	Single seropositive (*n* = 12, 21.4%)	Double seropositive (*n* = 13, 23.2%)	*P* between groups
Age—yr	58 (53.2–68.7)	58 (52.0–70.0)	56 (49.6–59.2)	61 (55.5–70.0)	0.250
Male—no. (%)	46 (82.1)	24 (77.4)	11 (91.7)	11 (84.6)	0.651
Smoking—no. (%)	22 (39.3)	14 (45.2)	4 (33.3)	4 (30.8)	0.847
Diabetes mellitus—no. (%)	6 (10.7)	4 (12.9)	0 (0)	2 (15.4)	0.584
Hypertension—no. (%)	19 (33.9)	12 (38.7)	1 (8.3)	6 (46.1)	0.202
Dyslipidemia—no. (%)	15 (26.8)	12 (38.7)	1 (8.3)	2 (15.4)	0.275
History of CAD—no. (%)	4 (7.1)	4 (12.9)	0 (0)	0 (0)	0.451
Anterior Infarction—no (%)	33 (58.9)	19 (61.3)	7 (58.3)	7 (53.8)	0.437
Hs-troponin at admission	3466 (282–11 094)	3558 (1593–11 065)	7227 (181–24 539)	216 (33–4708)	0.079
Peak hs-troponin I concentration—ng/L	77 428 (43 095–190 350)	53 846 (24 310–134 100)	130 500 (56 026–201 050)	109 200 (59 000–268 250)	0.067
Pain-to-balloon time—min	165.5 (115–395)	166 (115–424)	209 (157–411)	134 (112–156)	0.281
Post-PCI TIMI flow grade 3—no. (%)	8 (8.9%)	3 (9.7)	2 (16.6)	3 (23.1)	0.385
Echocardiographic parameters					
LVEDV—mL/m^2^	54.5 (47.2–63.7)	53 (45–61)	61.50 (50.571.5)	53 (51–57)	0.267
LVESV—mL/m^2^	27 (22–36.7)	25 (22–36.5)	29 (22.5–47.7)	29 (26–36)	0.528
LVEF—%	48.5 (40–55)	50 (40.7–54.2)	51 (33.2–56.7)	43 (37–51)	0.591
WMSI	1.71 (1.41–1–98)	1.71 (1.35–1.94)	1.73 (1.48–2.11)	1.76 (1.41–2)	0.591

CAD, coronary artery disease; hs, high sensitivity; LV, left ventricle; LVEDV, left ventricle end-diastolic volume; LVEF, left ventricle ejection fraction; LVESV, left ventricle end-systolic volume; PCI, percutaneous coronary intervention; WMSI, wall motion score index.

Looking at each seropositivity individually, MVO was more prevalent among AT1R-AAs seropositive patients [12/16 (75%)] than among AT1R-seronegative ones [11/40 (27.5%); *P* = 0.002]. Similarly, MVO was also more prevalent among ETAR-AAs seropositive patients [12/22 (54.5%)] compared to ETAR-AAs seronegative ones [11/34 (32.3%), *P* = 0.099]. The levels of both AT1R-AAs and ETAR-AAs were significantly higher in patients with MVO than in those without MVO (*Figure 1A*). For both AT1R-AAs and ETAR-AAs, there was a continuous nonlinear relationship between autoantibody levels and the probability of developing MVO (*Figure 1B*).

**Figure 1:**
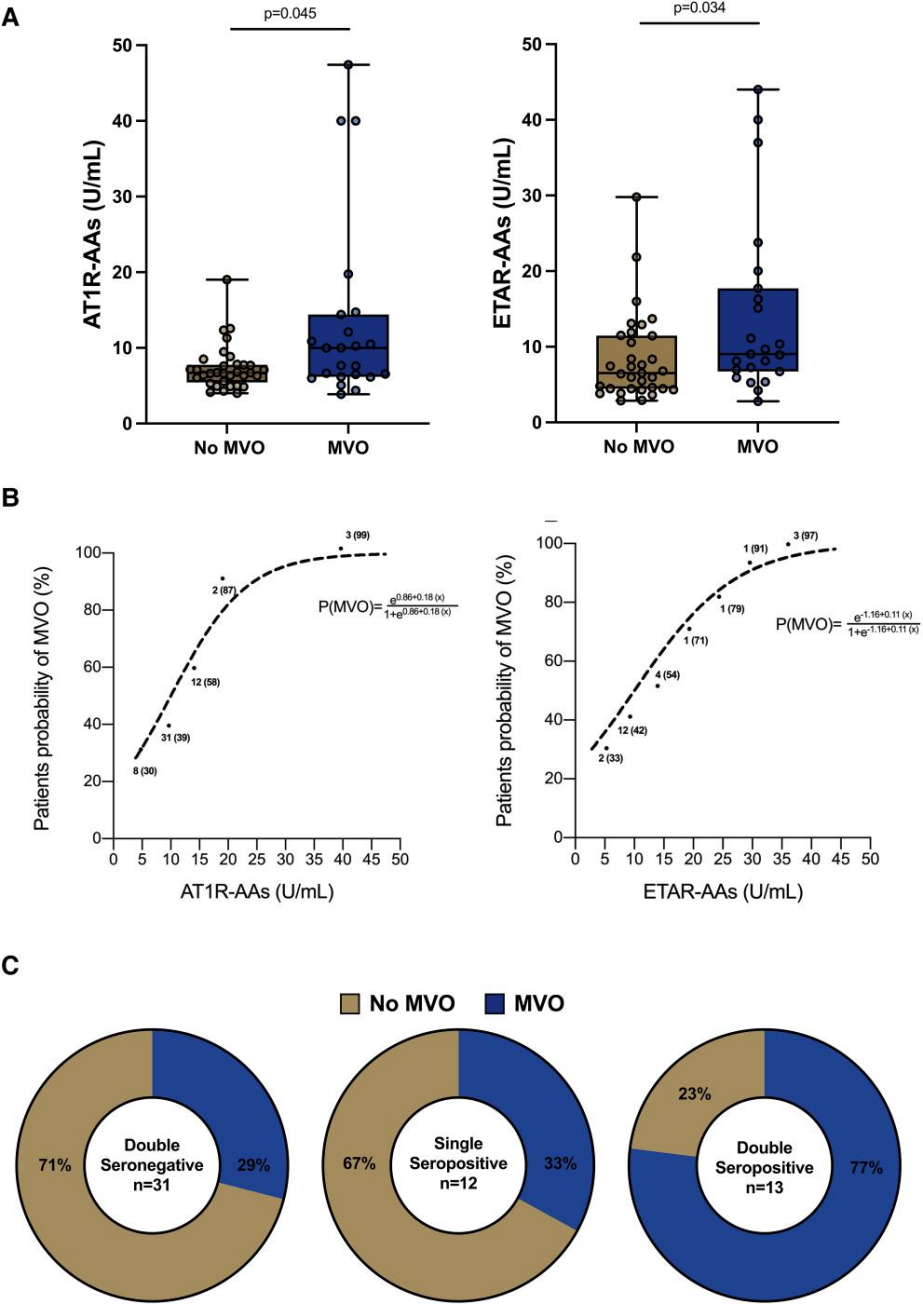
(*A*) Autoantibodies levels according to the presence of microvascular obstruction. Patients with microvascular obstruction have significantly higher levels of both autoantibodies targeting angiotensin II type 1 receptor and autoantibodies targeting endothelin 1 type A receptor compared to patients without microvascular obstruction. (*B*) Probability of microvascular obstruction according to continuous levels of autoantibodies. Logistic regression analysis revealed a continuous, nonlinear relationship between the levels of both autoantibodies targeting angiotensin II type 1 receptor and autoantibodies targeting endothelin 1 type A receptor and the probability of microvascular obstruction. (*C*) Prevalence of microvascular obstruction across the degrees of seropositivity. Patients with double seropositivity have a significantly greater prevalence of microvascular obstruction than patients with double seronegativity or single seropositivity (*P* between groups = 0.012). AT1R-AAs, autoantibodies targeting angiotensin II type 1 receptor; ETAR-AAs, autoantibodies targeting endothelin 1 type A receptor; MVO, microvascular obstruction.

Regarding the degree of seropositivity, 31 (55.4%) patients were double seronegative, 12 (21.4%) were single seropositive, and 13 (23.2%) were double seropositive. Among the 12 single seropositive patients, nine were positive for ETAR-AAs and three were positive for AT1R-AAs. There was a significant difference in the prevalence of MVO across the different degrees of autoantibody seropositivity (*P* = 0.012; *Figure 1C*). In detail, among patients with double seropositivity, the prevalence of MVO was significantly higher than that in double seronegative (*P* = 0.003) or single seropositive (*P* = 0.047) patients.

Recently, our group described the association between ETAR-AAs and MVO in patients with STEMI treated with PPCI.^2^ While in our previous study, we did not investigate the role of AT1R-AAs on MVO, from a pathophysiological point of view both ETAR-AAs and AT1R-AAs could be associated with MVO, through activation of similar molecular pathways and the ability to crosslink target receptors.^3^ Previous studies also support a possible association between AT1R-AAs and MVO: (i) their association with LVR after STEMI,^4^ a phenomenon that is highly correlated with MVO;^6^ (ii) their involvement in vascular rejection after organ transplantation, a condition that shares with MVO many histological features.^7,8^

To overcome the issues of the similarity of the effects and of the high degree of correlation between the two autoantibodies, we took a unique approach by assessing the degree of autoantibody seropositivity. Thus, we did not examine the effect of a single autoantibody but rather investigated the overall burden of anti-AT1R/ETAR autoimmunity. We hypothesize that the simultaneous presence of both autoantibodies exerts a larger effect by activating similar complementary intracellular pathways, which amplify their effects in a sort of ‘double hit’ phenomenon. This assumption is based on previous findings in other fields of medicine.^9^ For example, in preeclampsia, all patients have high levels of AT1R-AAs, but only those with severe disease are seropositive for ETAR-AAs.^10^ Based on these and other previous findings, we believe that the simultaneous presence of both autoantibodies is associated with a higher probability of MVO due to the amplification of the effects elicited by each single autoantibody. To confirm our hypothesis, further preclinical research is needed to better understand the molecular mechanisms through which AT1R-AAs and ETAR-AAs interact with each other and associate with MVO.

In conclusion, our study showed an association between the degree of seropositivity for AT1R-AAs/ETAR-AAs and MVO, suggesting a possible cumulative effect of these two autoantibodies on the risk of developing microvascular damage after STEMI. These findings could be promising for the development of new therapies for MVO.

## Data Availability

The data that support the findings of this study are available from the corresponding author, F.T., upon reasonable request.

## Lead author biography

Giovanni is a cardiologist and currently a PhD student at the University of Padua. Clinically, he is interested in heart failure, cardiac involvement of systemic diseases, and coronary microcirculation. His research interest also includes microvascular dysfunction after acute coronary syndromes and the interplay between mental stress and cardiovascular risk.
